# Long-Term Clinical Outcome of Cardiogenic Shock Patients Undergoing Impella CP Treatment vs. Standard of Care

**DOI:** 10.3390/jcm9123803

**Published:** 2020-11-24

**Authors:** Clemens Scherer, Enzo Lüsebrink, Danny Kupka, Thomas J. Stocker, Konstantin Stark, Christopher Stremmel, Mathias Orban, Tobias Petzold, Antonia Germayer, Katharina Mauthe, Stefan Kääb, Julinda Mehilli, Daniel Braun, Hans Theiss, Stefan Brunner, Jörg Hausleiter, Steffen Massberg, Martin Orban

**Affiliations:** 1 Intensive Care Unit and Department of Cardiology, Medizinische Klinik und Poliklinik I, Klinikum der Universität München, 81377 Munich, Germany; clemens.scherer@med.uni-muenchen.de (C.S.); e.luesebrink@med.uni-muenchen.de (E.L.); danny.kupka@med.uni-muenchen.de (D.K.); thomas.stocker@med.uni-muenchen.de (T.J.S.); konstantin.stark@med.uni-muenchen.de (K.S.); christopher.stremmel@med.uni-muenchen.de (C.S.); mathias.orban@med.uni-muenchen.de (M.O.); tobias.petzold@med.uni-muenchen.de (T.P.); antonia.germayer@med.uni-muenchen.de (A.G.); katharina.mauthe@med.uni-muenchen.de (K.M.); stefan.kaab@med.uni-muenchen.de (S.K.); julinda.mehilli@med.uni-muenchen.de (J.M.); daniel.braun@med.uni-muenchen.de (D.B.); Hans.Theiss@med.uni-muenchen.de (H.T.); stefan.brunner@med.uni-muenchen.de (S.B.); joerg.hausleiter@med.uni-muenchen.de (J.H.); steffen.massberg@med.uni-muenchen.de (S.M.); 2 DZHK (German Center for Cardiovascular Research), Partner Site Munich Heart Alliance, Medizinische Klinik und Poliklinik I, Klinikum der Universität München, 81377 Munich, Germany

**Keywords:** cardiogenic shock, mechanical circulatory support, Imella

## Abstract

The number of patients treated with the mechanical circulatory support device Impella Cardiac Power (CP) for cardiogenic shock is steadily increasing. The aim of this study was to investigate long-term survival and complications related to this modality. Patients undergoing Impella CP treatment for cardiogenic shock were retrospectively enrolled and matched with cardiogenic shock patients not treated with mechanical circulatory support between 2010 and 2020. Data were collected from the cardiogenic shock registry of the university hospital of Munich (DRKS00015860). 70 patients with refractory cardiogenic shock without mechanical circulatory support were matched with 70 patients treated with Impella CP. At presentation, the mean age was 67 ± 15 years with 80% being male in the group without support and 67 ± 14 years (*p* = 0.97) with 76% being male (*p* = 0.68) in the group with Impella. There was no significant difference in the rate of cardiac arrest (47% vs. 51%, *p* = 0.73) and myocardial infarction was the predominant cause of cardiogenic shock in both groups (70% vs. 77%). A total of 41% of patients without cardiocirculatory support and 54% of patients with Impella support died during the first month (*p* = 0.17). After one year, mortality rates were similar in both groups (55% in conventional vs. 59% in Impella CP group, *p* = 0.30) as was mortality rate at long-term 5-years follow-up (64% in conventional vs. 73% in Impella CP group, *p* = 0.33). The rate of clinically significant bleedings during ICU stay was lower in the conventional group than in the Impella support group (15% vs. 43%, *p* = 0.002). In this small observational and non-randomized analysis no difference in long-term outcome between patients treated with Impella CP vs. guideline directed cardiogenic shock therapy without mechanical circulatory support could be detected. Care must be taken regarding the high rate of bleeding and vascular complications when using Impella CP. Large, adequately powered studies are urgently needed to investigate the efficacy and safety of Impella CP in cardiogenic shock.

## 1. Introduction

Cardiogenic shock is still the major driver of mortality in cardiac intensive care units (ICU) and complicates around 10% of myocardial infarctions with contemporary 30-day and 1-year mortality up to 52% and 57% according to the latest large scale randomized trials (IABP II [1,2], CULPRIT-SHOCK trial [3,4]). Contemporary registries reported mortality rates ranging from 45–70% [5,6]. Cardiogenic shock as defined by the European Society of Cardiology (ESC) is specified by hypotension despite adequate filling status (systolic blood pressure <90 mmHg) with signs of hypoperfusion (clinical: cold sweated extremities, oliguria, mental confusion, dizziness, narrow pulse pressure; laboratory: metabolic acidosis, elevated serum lactate, elevated serum creatinine) due to primary cardiac dysfunction [7,8]. A significant subgroup of cardiogenic shock patients cannot be stabilized by guideline-directed volume and medical treatment alone. Here, mechanical circulatory support devices, in particular venoarterial extracorporeal membrane oxygenation (VA-ECMO) and Impella Cardiac Power (CP) transvalvular microaxial flow pump (Abiomed, Danvers, Massachusetts) [9] have emerged as established interventions [10,11,12].

The numbers of patients undergoing Impella CP treatment for cardiogenic shock rapidly increased during the last years due to its broad availability and easiness of insertion. However, there is only one small prospective randomized trial, the IMPRESS-IN-SEVERE-SHOCK trial which compared efficacy and safety of the Impella CP vs. intra-aortic balloon pump (IABP) in patients with acute myocardial infarction complicated by cardiogenic shock (AMICS) and did not show any survival benefit in the 48 patients included [13]. Numerous retrospective trials investigated acute and few mid-term outcomes of cardiogenic shock patients treated with Impella with maximum follow-up of 1 year [14] with 30-day mortality rates ranging from 36–52% [14,15,16,17]. Studies were also mixing different types of Impella, namely Impella 2.5, CP and 5.0 [18,19,20].

Hence, data on long-term outcome of cardiogenic shock patients exclusively undergoing Impella CP treatment are sparse and the first adequately powered randomized, multicenter Danish-German Cardiogenic Shock trial (NCT01633502) comparing Impella treatment with conventional guideline treatment alone [21] is ongoing and still far from finalization.

## 2. Methods

### 2.1. Study Population

In compliance with the Declaration of Helsinki and German data protection laws, cardiogenic shock patients treated in cardiac intensive care unit (ICU) of two Ludwig-Maximilians-University (LMU) hospitals, Campus Großhadern and Campus Innenstadt, between 2010 and 2020 were included in a registry (LMUshock). The latter is registered at the WHO International Clinical Trials Registry Platform (DRKS00015860) and was approved by the local ethics committee (IRB number: 18-001). The inclusion criterion of this registry was cardiogenic shock according to the IABP-SHOCK II trial definition: hypotension despite adequate filling status (systolic blood pressure <90 mmHg for 30 min or catecholamines to maintain systolic blood pressure >90 mmHg), clinical pulmonary congestion and impaired end-organ perfusion with at least one of the following criteria: altered mental status, cold, clammy skin and extremities, oliguria, elevated serum lactate level due to primary cardiac dysfunction [22]. For this study, patients (a) with ECMO- or IABP-treatment, (b) undergoing Impella support for high risk percutaneous coronary intervention (PCI), so called “protected PCI”, (c) undergoing treatment with Impella 2.5 or 5.0, (d) pericardial tamponade as cause of cardiogenic shock and (e) with missing data were excluded from this analysis. To adjust for confounders, patients with Impella CP support were matched by propensity score with patients without Impella CP support in a 1:1 ratio. Following patient consent, phone calls were performed to collect post-discharge clinical endpoints.

### 2.2. Patient Management during Impella Treatment

Details on Impella CP implantation have been reported previously [13]. Briefly, Impella CP implantation was exclusively performed in the catheterization laboratory under fluoroscopic control. The Impella CP catheter is a micro-axial continuous flow rotor pumps that can provide forward flow of up to 3.7 l/min. The device is recommended for insertion via femoral artery. A pigtail catheter is inserted into the left ventricle (LV) followed by introduction of a 0.18-inch Platinum Plus 260 cm guidewire (Boston Scientific, Natick, MA, USA) into LV to exchange the Impella CP catheter. The Impella is advanced over the guidewire into LV, wire is removed, and device support is initiated using the automated Impella Controller. Thereafter rotor speed is adjusted automatically to provide optimal forward flow or can be adjusted manually to desired performance level. Unfractionated heparin was intravenously administered to achieve an activated partial thromboplastin time above 60 s in the absence of bleeding complications. All patients were weaned at the discretion of the attending physician. Impella CP decannulation was routinely performed at bedside with compression of the arterial access site using a compression system.

### 2.3. Study Endpoints

Ischemic (death, myocardial infarction, stent thrombosis, stroke, vascular complications) and bleeding events (classified according to the bleeding academic research consortium (BARC)) [23] during hospital stay and at the latest possible follow-up were obtained and analyzed. To evaluate the neurologic outcome Pittsburgh Cerebral Performance Category (CPC) at ICU discharge was determined.

### 2.4. Statistical Analysis

Statistical analysis was performed using R (version 4.0.1, The R foundation, Vienna, Austria). Normally distributed continuous variables were reported as mean with standard deviation and non-normally distributed continuous variables as median with interquartile ranges (25th and 75th percentile). To compare two groups, paired and unpaired t-test for normally distributed continuous variables and paired and unpaired Mann-Whitney-U test for non-normally distributed continuous variables were used. Categorical variables were reported as absolute numbers and percentages. To compare unpaired groups, chi-squared test was utilized. For matched paired groups, McNemar’s test was used. All tests were 2-tailed, and *p*-values < 0.05 were considered as significant. Mortality and bleeding rates were calculated using the Kaplan–Meier method and comparisons were made by using log-rank tests. For propensity score matching, the R package “MatchIt” version 3.0.2 was utilized with a 1:1 nearest neighbor algorithm, no replacement, logistic link distance measure and a caliper of 0.1 [24]. The following baseline parameters which are known to impact ICU mortality in cardiogenic shock were used for matching: Age, gender, first measured lactate measured at ICU admission, ventilation on ICU, myocardial infarction at presentation and cardiac arrest [16,25]. The propensity score was estimated by logistic regression. After matching standard difference of mean was below 0.1 for all parameters. To assess the correlation of clinical and laboratory parameters with mortality, univariate and multivariate Cox proportional hazard models were used. Covariates included age, diabetes, hypertension, first measured lactate on ICU, first measured GFR on ICU, cardiac arrest, Impella treatment and myocardial infarction. Stepwise selection of parameters for multivariate analysis was performed by Akaike information criterion (AIC) with backward direction and 1000 bootstrap iterations using the stepAIC function of the R package MASS (version 7.3-51.6).

## 3. Results

### 3.1. Study Population and Baseline Characteristics

**Figure 1:**
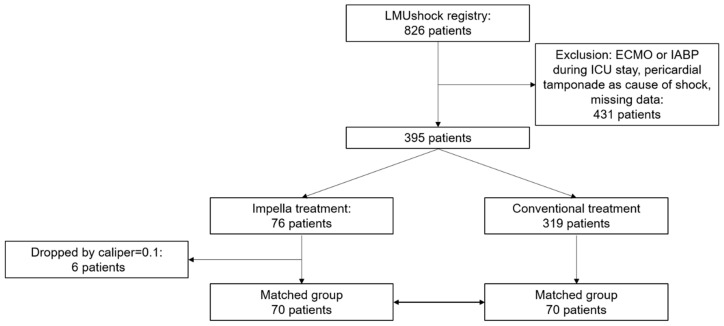
Study Flow chart. Flow diagram depicting patient selection.

Registry data of 826 patients with cardiogenic shock treated in two cardiologic ICUs between 2010 and 2020 were available. After exclusion of patients according to exclusion criteria, 76 patients with Impella support were considered eligible for matching (Study Flow chart, Figure 1). Due to six missing matching partners when using a caliper of 0.1 for matching, 70 patients with Impella support were matched with 70 patients without mechanical circulatory support as described above, achieving a standard difference of mean below 0.1 for all matching parameters (Supplementary Figure S1).

At presentation, the mean age was 67 ± 15 years with 80% being male in the group without support and 67 ± 14 years (*p* = 0.97) with 76% being male (*p* = 0.68) in the group with Impella. Cardiovascular diseases and cardiovascular risk factors before onset of cardiogenic shock were distributed evenly. There was no significant difference in the rate of cardiac arrest (47% vs. 51%, *p* = 0.73), duration of cardio-pulmonary resuscitation if applicable [minutes] (median 24.0, interquartile range (IQR) (9.5,52.5) vs. 19.0 (1.0,26.5), *p* = 0.22). Myocardial infarction was the predominant cause of cardiogenic shock in both groups (70% vs. 77%). The baseline characteristics of both groups are displayed in Table 1. Patients’ characteristics before matching are shown in Supplementary Table S1.

### 3.2. ICU Parameters

The SAPS-II scores (median 68.6, IQR (58.2,76.0) vs. 68.6 (62.5,76.8), *p* = 0.53) were almost identically between both groups. The median length of ICU stay was shorter in the conventional as compared to the Impella group [days] (median 3.5, IQR (1.2,8.7) vs. 5.5 (1.9,12.5), *p* = 0.04). There was no difference in the rate of mechanical ventilation (74% vs. 77%, *p* = 0.82). Systolic blood pressure was similar [mmHg] (median 108, IQR (101,117) vs. 107 (101,114), *p* = 0.67) in both groups. Patients in the control group had a significant lower diastolic blood pressure [mmHg] (median 57, IQR (50,60) vs. 60 (57,66), *p* < 0.01) and a lower average heart rate [bpm] (median 84, IQR (77,92) vs. 91 (83,101), *p* < 0.01). The ICU parameters for both groups are displayed in Table 2. Medication at ICU discharge is displayed in Supplemental Table S2. Lactate levels are detailed in Supplementary Figures S2 and S3.

### 3.3. Clinical Endpoints

**Figure 2:**
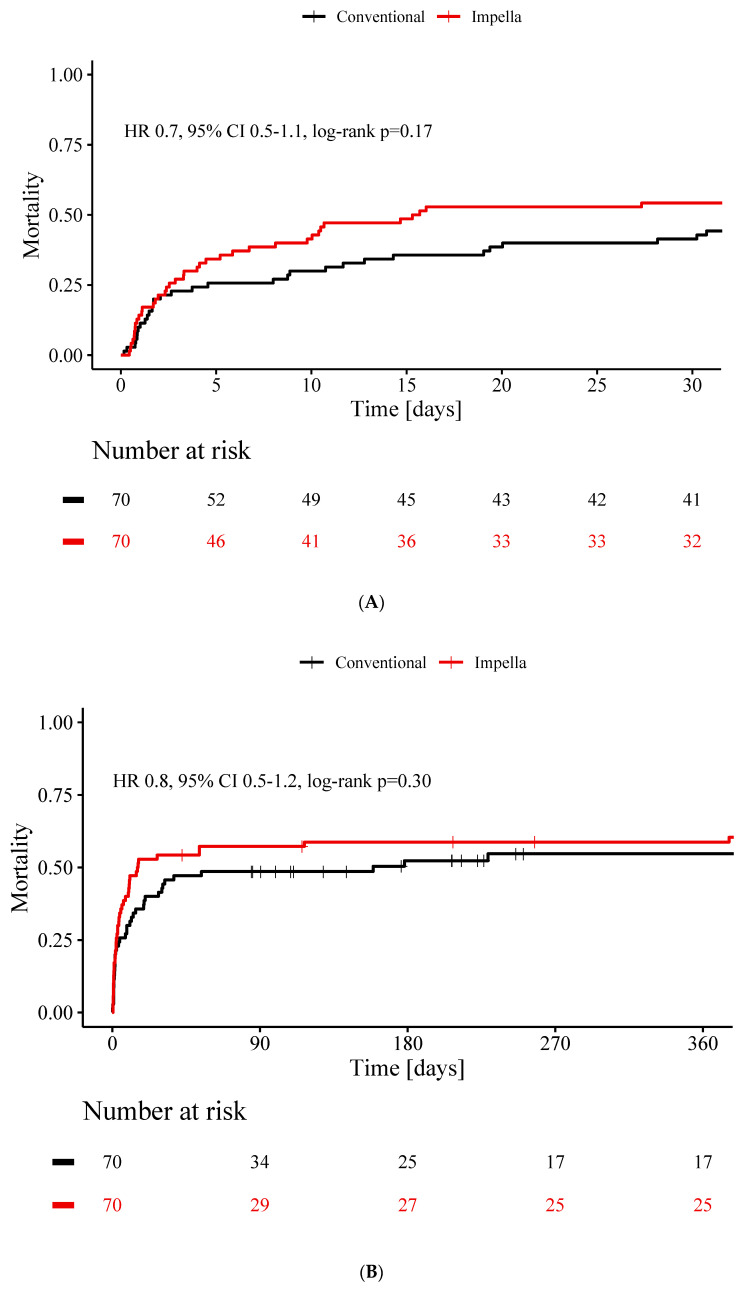

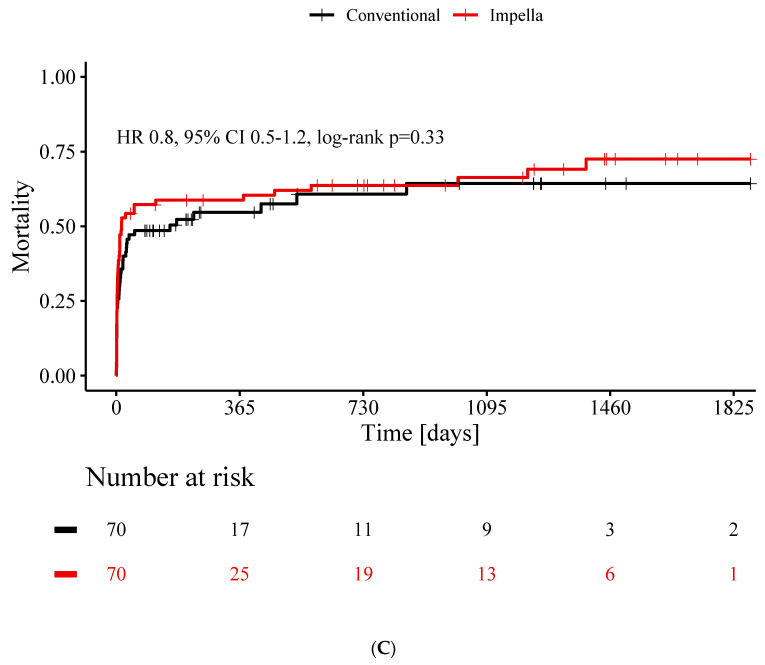
Mortality of patients with cardiogenic shock treated without cardiocirculatory support versus patients treated with Impella CP. Cumulative incidence curves of deaths for patients without cardiocirculatory support (black) and with Impella support (red) 30 days (**A**), 1 year (**B**) and 5 years (**C**) after the index event. Hazard ratio and 95% confidence interval is displayed for conventional group. HR, hazard ratio; CI, confidence interval.

A total of 41% of patients without cardiocirculatory support and 54% of patients with Impella support died during the first month (*p* = 0.17) (Figure 2A). After one year, mortality rates were similar in both groups (55% in conventional vs. 59% in Impella CP group, *p* = 0.30) (Figure 2B) as was mortality rate at long-term 5-years follow-up (64% in conventional vs. 73% in Impella CP group, *p* = 0.33) (Figure 2C).

**Figure 3:**
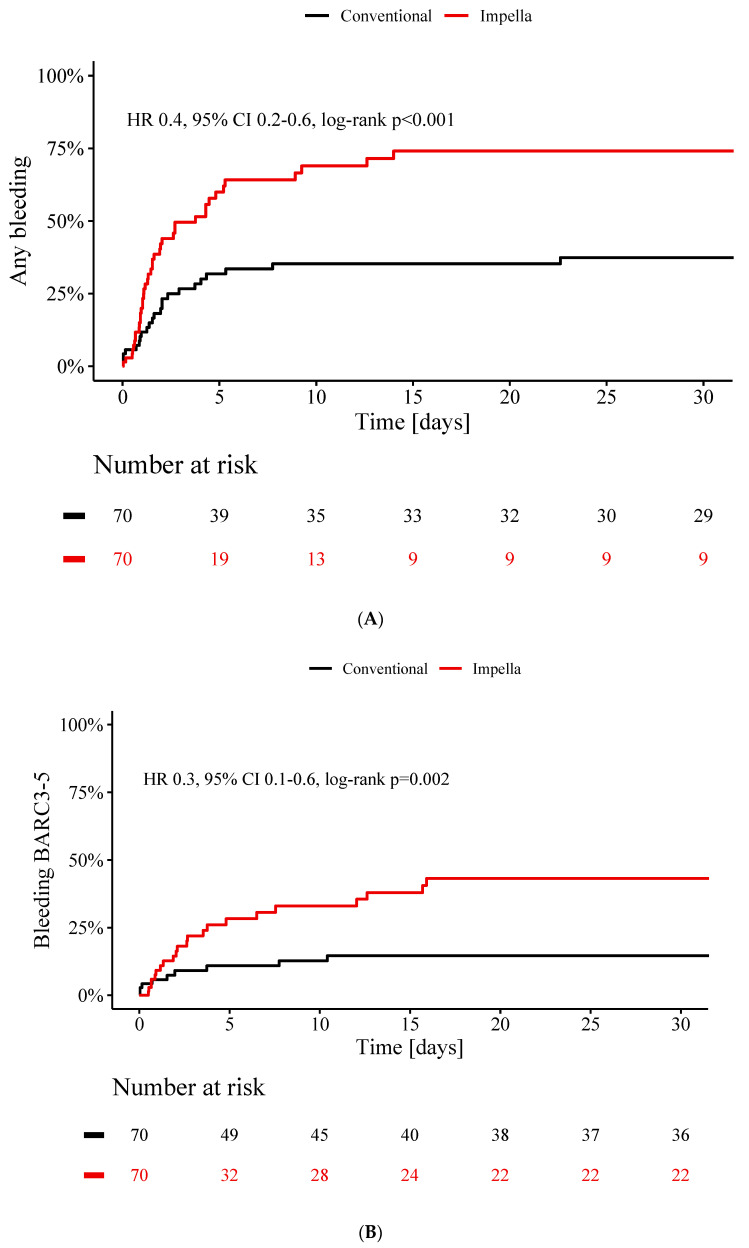
Bleeding complications in patients treated without cardiocirculatory support versus patients treated with Impella CP. (**A**), Cumulative incidence curves of all bleedings occurring in patients without (black) and with Impella CP (red) are shown for 30 days after the index event. (**B**), Cumulative incidence curves of bleeding complications at least classified as BARC3 occurring in patients without (black) and with Impella CP (red) are shown for 30 days after the index event. HR and 95% CI is displayed for conventional group.

The rate of any bleeding event during ICU stay (37% vs. 74%, *p* < 0.001) (Figure 3A) as well as the rate of clinically significant bleedings classified as BARC 3 or higher was lower in the conventional group than in Impella CP group (15% vs. 43%, *p* = 0.002) (Figure 3B). There were no vascular complications in the control group whereas 7% of the patients in the Impella group (*p* = 0.07) suffered from vascular complications.

**Figure 4:**
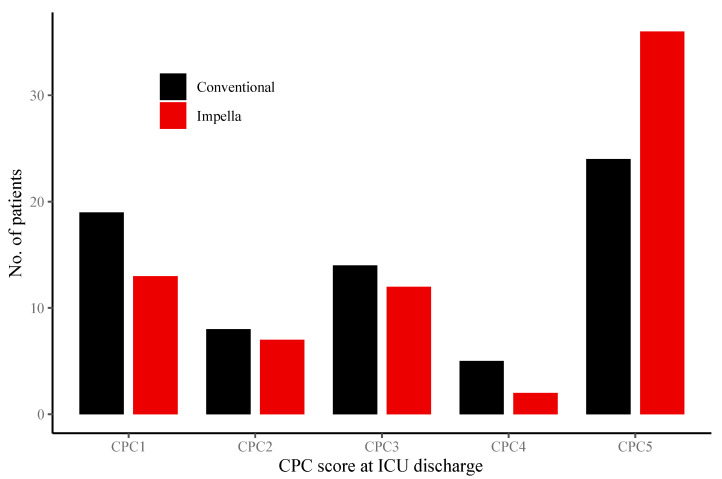
Neurologic outcome of patients treated without cardiocirculatory support versus patients treated Impella support. CPC scores at discharge of patients with no cardiocirculatory support (black) versus Impella support (red) (*p* = 0.28) are displayed.

The neurologic outcome, measured as CPC score at discharge from ICU, was similar in both groups (*p* = 0.28) (Figure 4). All clinical endpoints are shown in Table 3.

In a Cox proportional hazard regression on long term-survival, we identified age, first measured lactate, first measured GFR on ICU and cardiac arrest as independent risk factors (Table 4).

## 4. Discussion

This study including cardiogenic shock patients treated with standard of care vs. mechanical circulatory support using Impella CP between 2010 and 2020 investigated long-term outcome and bleeding complications. The main findings of our study are as follows: (1) There was no statistically significant difference in 30-day, 1-year and 5 years mortality rate between both groups, (2) Impella CP treatment was accompanied with an almost tripled rate of clinical significant bleedings compared to the control group, and (3) the median length of ICU-stay was longer in the Impella CP group.

To the best of our knowledge, we here report the first investigation on long-term mortality of cardiogenic shock patients treated with standard of care vs. mechanical circulatory support using Impella CP. Several retrospective studies and large registries did not focus on cardiogenic shock including mostly patients in which Impella was used as “protection” device in high risk PCI [15]. And yet others did not focus on Impella CP by including different types of devices, namely Impella 2.5, CP, 5.0 and even Tandem Heart within their analysis [17,18,19,20,26,27,28]. In this regard, the Impella 2.5 is limited by its pump volume of only 2.5 l/min compared to the contemporary available CP and CP Smart Assist which are able to pump up to 3.5 to 4.0 l/min. The drawback of Impella 5.0 is the need for surgical cutdown which largely limits its use in a salvage or emergency setting. In our analysis we exclusively focused on both, Impella CP and cardiogenic shock, and excluded patients undergoing concomitant VA-ECMO treatment [11].

In others trials—focusing on Impella CP and cardiogenic shock—mid-term follow-up is limited to a maximum between 70 days and 12 month [14,18,19]. Here, we expand the available follow up-time up to 5 years.

Our 30-day and 1-year mortality rates were 54% and 59% in the Impella group, respectively. This is slightly higher compared to the small randomized IMPRESS trial (24 patients on Impella) which compared treatment with Impella CP vs. intra-aortic balloon pump [13]) with reported 30-day mortality of 46% which might be attributed to the fact that our analysis is an all-comers trial. Our mortality rate is lower compared to Loehn et al. (73 patients on Impella CP) [14], who reported mortality at discharge of 64%. Interestingly, in the latter trial pre-PCI implanted Impella CP treated patients showed a lower mortality rate of 50% compared to post-PCI Impella CP implantation who showed a much higher mortality rate of 77%. In our center implantation of Impella CP was always performed post-PCI.

There is an ongoing debate if pre-implantation of Impella can reduce infarct size as shown in the Door-To-Unload in STEMI Pilot Trial (DTU-STEMI) [29]. In this trial, the authors compared a 30 min delay for LV-unloading implanting the Impella CP device before revascularization vs. Impella implantation followed by immediate revascularization. However, patients in the latter trial did not suffer from cardiogenic shock and the study lacked a standard of care arm. If this new concept of care can possibly improve outcome is currently tested in the ongoing prospective, multicenter, randomized, controlled open-label two-arm Primary Unloading and Delayed Reperfusion in ST-Elevation Myocardial Infarction: The STEMI-DTU Trial (NCT03947619). This trial might answer the question if a postponed re-vascularization of infarct related artery will cause harm. By now, our strategy of post-PCI implantation shows a mortality rate at 30-days (54%) which is comparable to the pre-PCI implantation cohort of the study of Loehn et al. (52%) [14].

Bleeding complication occur frequently with Impella treatment and were reported as high as in our trial [16]. Using such devices—with an almost mandatory need for therapeutic anticoagulation—require a constant vigilance to prevent severe bleedings associated with access site puncture. Hence, the high rate of bleeding complication remains one of the most important disadvantages of this device.

Interestingly, we observed a longer ICU stay (plus 2 days) in patients undergoing Impella CP treatment although the SAPS II score at admission, the rate of mechanical ventilation and cardiac arrest was well matched between groups. This longer treatment period does not translate in improvement of in-hospital and 30-day or long-term survival but might instead increase treatment costs of this already very expensive treatment strategy. Since most trials investigating Impella treatment lack a control group without any mechanical support, the prolonged ICU stay of the Impella CP group is a new finding. In the setting of this trial factors like left ventricular and neurologic function might have a greater impact on long term outcome compared to temporary circulatory support.

Mechanical circulatory support using Impella CP has still not been investigated in large, randomized trials. Hence, the results of the randomized, multicenter Danish-German Cardiogenic Shock trial (NCT01633502) are urgently needed to prove the efficacy in reducing mortality of this very expensive device. Furthermore, future analysis should aim at investigating the role of Impella CP as bridge to recovery, left ventricular assist devices and heart transplantation in patients in cardiogenic shock class B and C according to SCAI definition [30]. This study did not compare Impella CP vs. VA-ECMO treatment for cardiogenic shock. In contrast to Impella CP, VA-ECMO treatment is restricted to a limited number of tertiary care centers in many countries and according to the latest update on cardiogenic shock published in the European Heart Journal last year [10] - this device is rather used for treatment of most severe cardiogenic shock Type E defined by lactate > 5mmol/L [30]. Correspondingly, median lactate on admission in ICU in both groups was 3 mmol/L.

Limitations: This retrospective, observational, analysis investigates mechanical circulatory support with Impella CP in a small patient cohort, but is the first analysis investigating long-term follow up in cardiogenic shock patients and presents a control group without any mechanical circulatory support. The indication of implanting a mechanical support device was at the physician’s discretion. We acknowledge that matching of groups from different treatment periods comes along with limitations inherent to such analysis. Differences between the two groups may not have been detectable due to the small sample size and the inherent heterogeneity of patients treated with Impella CP. Matching of patients can only decrease but not abrogate confounding factors, which may bias the results of this study. This study did not compare Impella CP vs. VA-ECMO treatment for cardiogenic shock.

## 5. Conclusions

In this small observational and non-randomized analysis no difference in long-term outcome between patients treated with Impella CP vs. guideline directed cardiogenic shock therapy without mechanical circulatory support could be detected. Care must be taken regarding the high rate of bleeding and vascular complications when using Impella CP. Large, adequately powered studies are urgently needed to investigate the efficacy and safety of Impella CP in cardiogenic shock.

## Figures and Tables

**Table 1:** Baseline characteristics of matched patients.

Variables	Conventional (*n* = 70)	Impella (*n* = 70)	*p* Value (Paired)	*p* Value (Not Paired)
Age in years, mean (SD)	67.3 (14.8)	67.2 (14.1)	0.97	0.97
Male gender, *n* (%)	56 (80.0)	53 (75.7)	0.68	0.68
Body mass index, mean (SD)	27.6 (5.6)	27.3 (4.5)	0.79	0.77
Coronary artery disease, *n* (%)	41 (58.6)	47 (67.1)	0.39	0.38
Previous myocardial infarction, *n* (%)	23 (32.9)	17 (24.3)	0.36	0.35
Previous PCI, *n* (%)	22 (31.4)	25 (35.7)	0.74	0.72
Previous CABG, *n* (%)	4 (5.7)	2 (2.9)	0.68	0.68
Atrial fibrillation, *n* (%)	25 (35.7)	20 (28.6)	0.49	0.47
Previous stroke, *n* (%)	8 (11.4)	9 (12.9)	1.00	1.00
Peripheral artery disease, *n* (%)	17 (24.3)	12 (17.1)	0.46	0.40
Smoker, *n* (%)	-	-	0.95	0.94
Active smoker	16 (22.9)	17 (24.3)		
Former smoker	16 (22.9)	17 (24.3)		
Never smoked	38 (54.3)	36 (51.4)		
Hypertension, *n* (%)	51 (72.9)	48 (68.6)	0.71	0.71
High cholesterol, *n* (%)	42 (60.0)	32 (45.7)	0.11	0.13
Diabetes, *n* (%)	23 (32.9)	23 (32.9)	1.00	1.00
Positive cardiovascular family history, *n* (%)	8 (11.4)	11 (15.7)	0.63	0.62
Simplified acute Physiology Score II-score, median (IQR)	68.6 (58.2,76.0)	68.6 (62.5,76.8)	0.53	0.45
Cardiac arrest, *n* (%)	33 (47.1)	36 (51.4)	0.73	0.74
Out of hospital cardiac arrest, *n* (%)	21 (30.0)	14 (20.0)	0.21	0.24
Duration of cardio-pulmonary resuscitation if applicable in minutes, median (IQR)	24.0 (9.5,52.5)	19.0 (1.0,26.5)	NA	0.22
Cause of cardiogenic shock, *n* (%)	-	-	NA	0.38
STEMI	25 (35.7)	28 (40.0)		
NSTEMI	24 (34.3)	26 (37.1)		
Cardiomyopathy	13 (18.6)	13 (18.6)		
Myocarditis	1 (1.4)	2 (2.9)		
Arrhythmia	3 (4.3)	0 (0.0)		
Valvular	1 (1.4)	0 (0.0)		
Other	3 (4.3)	1 (1.4)		
PCI during hospital stay, *n* (%)	44 (62.9)	53 (75.7)	0.10	0.14
CABG during hospital stay, *n* (%)	1 (1.4)	0 (0.0)	1.00	1.00

**Table 2:** ICU parameters.

Variables	Conventional (*n* = 70)	Impella (*n* = 70)	*p* Value (Paired)	*p* Value (Not Paired)
Duration of ICU stay in days, median (IQR)	3.5 (1.2,8.7)	5.5 (1.9,12.5)	0.04	0.16
Average systolic blood pressure in mmHg, median (IQR)	107.7 (100.9,116.5)	107.4 (100.5,113.5)	0.67	0.57
Average diastolic blood pressure in mmHg, median (IQR)	56.6 (49.9,60.0)	59.5 (56.8,65.7)	<0.01	<0.01
Average heart rate in bpm, median (IQR)	84.4 (77.1,92.4)	91.2 (82.8,101.1)	<0.01	<0.01
First lactate on ICU in mmol/L, median (IQR)	3.5 (1.6,7.5)	3.4 (1.9,7.5)	0.82	0.72
First GFR on ICU in mL/min, median (IQR)	49.6 (30.2,60.0)	49.2 (37.2,60.0)	0.89	0.97
Mechanical ventilation, *n* (%)	52 (74.3)	54 (77.1)	0.82	0.84
ASS treatment, *n* (%)	47 (67.1)	43 (61.4)	0.64	0.60
Clopidogrel treatment, *n* (%)	30 (42.9)	34 (48.6)	0.62	0.61
Prasugrel treatment, *n* (%)	21 (30.0)	16 (22.9)	0.44	0.44
Ticagrelor treatment, *n* (%)	2 (2.9)	5 (7.1)	0.45	0.44

**Table 3:** Clinical endpoints.

Variables	Conventional (*n* = 70)	Impella (*n* = 70)	*p* Value (Paired)	*p* Value (Not Paired)
CPC on discharge, *n* (%)	-	-	NA	0.28
CPC1	19 (27.1)	13 (18.6)		
CPC2	8 (11.4)	7 (10.0)		
CPC3	14 (20.0)	12 (17.1)		
CPC4	5 (7.1	2 (2.9)		
CPC5	24 (34.3)	36 (51.4)		
Mortality rate after 30 days,% (95% CI)	41.4 (28.7, 51.9)	54.3 (41.0, 64.6)	0.17	
Mortality rate after one year,% (95% CI)	54.7 (40.7, 65.4)	58.8 (45.4, 68.9)	0.30	
Mortality rate after 5 years,% (95% CI)	64.4 (47.6, 75.8)	72.5 (56.9, 82.5)	0.33	
Bleeding rate ≥ BARC3,% (95% CI)	14.7 (5.2, 23.2)	43.1 (27.0, 55.7)	0.002	
Bleeding rate BARC1-5,% (95% CI)	37.3 (23.8, 48.5)	74.1 (58.1, 84.0)	<0.001	
Myocardial infarction during ICU stay, *n* (%)	3 (4.3)	1 (1.4)	0.62	0.61
Stroke during ICU stay, *n* (%)	1 (1.4)	4 (5.7)	0.37	0.36
Vascular complications during ICU stay, *n* (%)	0 (0.0)	5 (7.1)	0.07	0.07

**Table 4:** Cox proportional hazard regression for long term-survival.

Risk Factor	Univariate Analysis			Multivariate Analysis after Feature Selection		
	Hazard Ratio	95% CI	*p* Value	Hazard Ratio	95% CI	*p* Value
Age [years]	1.040	1.022–1.058	0.000	1.046	1.026–1.067	<0.001
Diabetes	1.216	0.786–1.880	0.380	-	-	-
Hypertension	1.642	1.005–2.685	0.048	-	-	-
First lactate measured on ICU [per mmol/L]	1.151	1.097–1.209	0.000	1.099	1.034–1.168	0.002
First GFR measured on ICU [per mL/min]	0.983	0.972–0.993	0.001	0.987	0.975–1.000	0.042
Cardiac arrest	1.915	1.250–2.932	0.003	1.887	1.096–3.249	0.022
Impella treatment	1.235	0.808–1.887	0.330	-	-	-
Myocardial infarction	1.374	0.826–2.287	0.221	-	-	-

## Supplementary Materials

The following are available online at https://www.mdpi.com/2077-0383/9/12/3803/s1, Figure S1: Love plot. Love plot showing standardized mean differences of unadjusted parameters before and adjusted parameters after matching; Figure S2: Lactate levels. Lactate levels after ICU admission in patients treated without cardiocirculatory support versus patients treated with Impella CP (day 1: *p* = 0.57, day 2: *p* = 0.91, day 3: *p* = 0.26, day 4: *p* = 0.36, day 5: *p* = 0.47); Figure S3. Lactate levels. Lactate levels in patients treated with Impella CP after implantation; Table S1. Patients’ characteristics before matching; Table S2. Medication at ICU discharge.
