# Supplementary material for: Long-Term Clinical Outcome of Cardiogenic Shock Patients Undergoing Impella CP Treatment vs. Standard of Care
Source: J Clin Med. 2020 Nov 24;9(12):3803. doi: 10.3390/jcm9123803 (PMC7760637; doi:10.3390/jcm9123803)
Supplement: Supplementary file 1 [file jcm-09-03803-s001.pdf]

Supplementary Materials

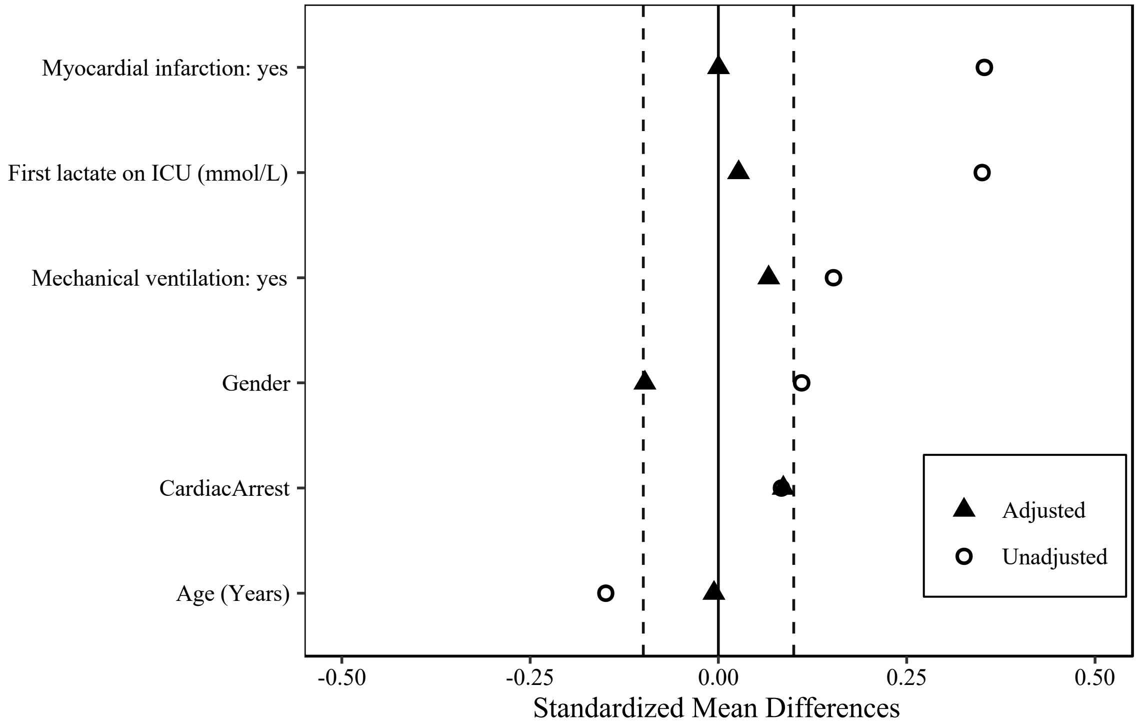

Supplementary Figure S1. Love plot. Love plot showing standardized mean differences of unadjusted parameters before and adjusted parameters after matching.

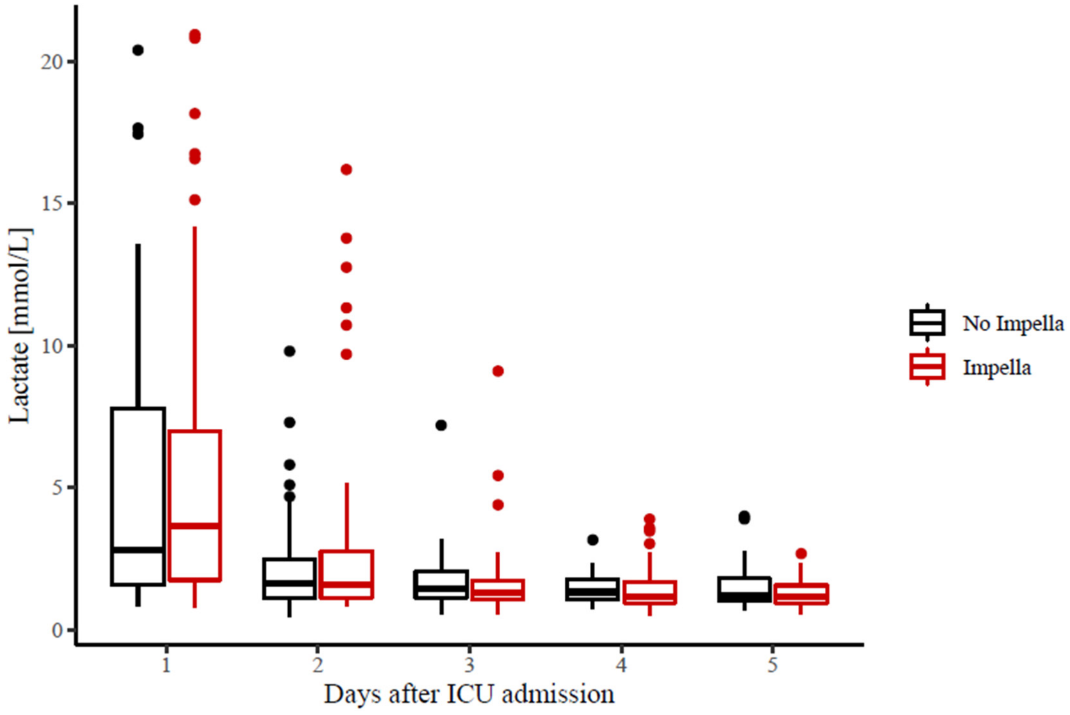

Supplementary Figure S2. Lactate levels. Lactate levels after ICU admission in patients treated without cardiocirculatory support versus patients treated with Impella CP (day 1: $p = 0.57$, day 2: $p = 0.91$, day 3: $p = 0.26$, day 4: $p = 0.36$, day 5: $p = 0.47$) .

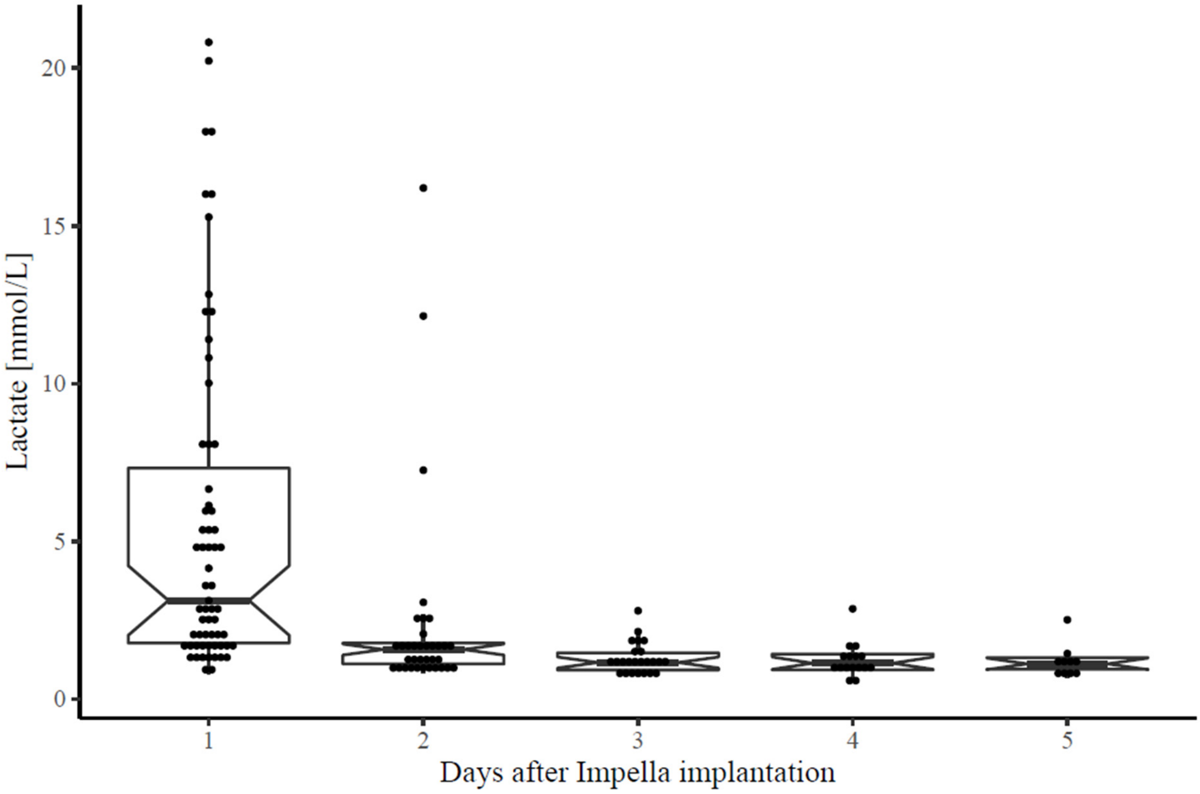

Supplementary Figure 3. Lactate levels. Lactate levels in patients treated with Impella CP after implantation.

Supplementary Table S1. Patients' characteristics before matching

**Variables**	**Conventional (*n* = 319)**	**Impella (*n* = 76)**	***p* value**
Age in years, mean (SD)	69.4 (14.2)	67.3 (14.2)	0.24
Male gender, *n* (%)	228 (71.5)	58 (76.3)	0.48
Body mass index, mean (SD)	27.3 (7.2)	27.3 (4.4)	0.94
Coronary artery disease, *n* (%)	200 (62.7)	52 (68.4)	0.42
Previous myocardial infarction, *n* (%)	91 (28.5)	19 (25.0)	0.64
Previous PCI, *n* (%)	97 (30.4)	27 (35.5)	0.47
Previous CABG, *n* (%)	16 (5.0)	2 (2.6)	0.56
Atrial fibrillation, *n* (%)	129 (40.4)	22 (28.9)	0.09
Previous stroke, *n* (%)	35 (11.0)	10 (13.2)	0.74
Peripheral artery disease, *n* (%)	66 (20.7)	13 (17.1)	0.59

Smoker, *n* (%)<br>Active smoker<br>Former smoker<br>Never smoked	 55 (17.2)<br>65 (20.4)<br>199 (62.4)	 20 (26.3)<br>17 (22.4)<br>39 (51.3)	0.13
Hypertension, *n* (%)	232 (72.7)	52 (68.4)	0.54
High cholesterol, *n* (%)	154 (48.3)	35 (46.1)	0.83
Diabetes, *n* (%)	108 (33.9)	23 (30.3)	0.64
Positive cardiovascular family history, *n* (%)	43 (13.5)	12 (15.8)	0.74
Simplified acute Physiology Score II-score, median [IQR]	68.9 [60.0, 78.0]	68.9 [62.8, 77.5]	0.59
Cardiac arrest, *n* (%)	163 (51.1)	42 (55.3)	0.60
Out of hospital cardiac arrest, *n* (%)	90 (28.2)	16 (21.1)	0.26
Duration of cardio-pulmonary resuscitation if applicable in minutes, median [IQR]	15.0 [5.0, 31.0]	20.0 [2.0, 28.0]	0.96
Cause of cardiogenic shock, *n* (%)<br>STEMI<br>NSTEMI<br>Cardiomyopathy<br>Myocarditis<br>Arrhythmia<br>Valvular<br>Other	 86 (27.0)<br>97 (30.4)<br>56 (17.6)<br>6 (1.9)<br>34 (10.7)<br>18 (5.6)<br>21 (6.6)	 30 (39.5)<br>29 (38.2)<br>13 (17.1)<br>2 (2.6)<br>0 (0.0)<br>0 (0.0)<br>2 (2.6)	0.01

Supplementary Table S2. Medication at ICU discharge

**Medication name**	**Conventional (*n* = 46)**	**Impella (*n* = 34)**	***p* value**
ASS, *n* (%)	35 (76.1)	24 (70.6)	0.77
Clopidogrel, *n* (%)	18 (39.1)	17 (50.0)	0.46
Prasugrel, *n* (%)	11 (23.9)	7 (20.6)	0.94
Ticagrelor, *n* (%)	1 (2.2)	2 (5.9)	0.79
Betablocker, *n* (%)	21 (45.7)	22 (64.7)	0.14
Statin, *n* (%)	30 (65.2)	18 (52.9)	0.38
